# SIRT2 Deficiency Exacerbates Hepatic Steatosis via a Putative Role of the ER Stress Pathway

**DOI:** 10.3390/ijms23126790

**Published:** 2022-06-17

**Authors:** Helena Leal, João Cardoso, Patrícia Valério, Marta Quatorze, Vítor Carmona, Janete Cunha-Santos, Luís Pereira de Almeida, Cláudia Pereira, Cláudia Cavadas, Pedro Gomes

**Affiliations:** 1 Center for Neuroscience and Cell Biology, University of Coimbra, 3004-504 Coimbra, Portugal; hleal@cnc.uc.pt (H.L.); jmcardoso@cnc.uc.pt (J.C.); patricia.valerio@unibas.ch (P.V.); marta.correia@cnc.uc.pt (M.Q.); vitor.carmona@cnc.uc.pt (V.C.); janetecunhasantos@gmail.com (J.C.-S.); luispa@ci.uc.pt (L.P.d.A.); cpereira@cnc.uc.pt (C.P.); 2 Center for Innovative Biomedicine and Biotechnology (CIBB), University of Coimbra, 3004-504 Coimbra, Portugal; 3 Faculty of Pharmacy, University of Coimbra, 3004-504 Coimbra, Portugal; 4 Faculty of Medicine, University of Coimbra, 3004-508 Coimbra, Portugal; 5 Department of Biomedicine, Faculty of Medicine, University of Porto, 4200-319 Porto, Portugal

**Keywords:** SIRT2, hepatic steatosis, insulin resistance, ER stress

## Abstract

Nonalcoholic fatty liver disease (NAFLD), a condition strongly associated with obesity and insulin resistance, is characterized by hepatic lipid accumulation and activation of the endoplasmic reticulum (ER) stress response. The sirtuin 2 (SIRT2) protein deacetylase is emerging as a new player in metabolic homeostasis, but its role in the development of hepatic steatosis and its link with ER stress activation remains unknown. SIRT2-knockout (SIRT2-KO) and wild-type mice were fed either a control or a high-fat diet (HFD) for 4 weeks. Genetic manipulation of SIRT2 levels was performed in human hepatic cells. Although apparently normal under a control diet, SIRT2-KO mice showed accelerated body weight gain and adiposity on a HFD, accompanied by severe insulin resistance. Importantly, SIRT2-KO mice exhibited worsened hepatic steatosis independently from diet, consistent with upregulated gene expression of lipogenic enzymes and increased expression of ER stress markers. Exposure of hepatic cells to palmitate induced lipid accumulation, increased ER stress, and decreased SIRT2 expression. Moreover, SIRT2-silenced cells showed enhanced lipid accumulation and ER stress activation under basal conditions, whereas SIRT2 overexpression abrogated palmitate-induced lipid deposition and ER stress activation. Our findings reveal a role for SIRT2 in the regulation of hepatic lipid homeostasis, potentially through the ER stress response, suggesting that SIRT2 activation might constitute a therapeutic strategy against obesity and its metabolic complications.

## 1. Introduction

Nonalcoholic fatty liver disease (NAFLD) is the most common liver disorder worldwide [1] and is tightly linked to obesity and insulin resistance [2,3,4]. NAFLD encompasses a progressive spectrum of histopathological conditions, in which the earliest stage and disease hallmark is characterized by free fatty acid overload in hepatocytes [5]. This, in turn, can lead to mitochondrial dysfunction and endoplasmic reticulum (ER) stress, which promote NAFLD progression [3]. With continued fat accumulation, susceptibility to hepatocyte injury and inflammation increases, leading to a condition termed nonalcoholic steatohepatitis (NASH), which can progress to cirrhosis and ultimately hepatocellular carcinoma [6]. Despite intensive research, fundamental gaps remain in our mechanistic understanding of NAFLD pathophysiology.

The ER stress response has emerged as a crucial player in fatty acid and cholesterol metabolism [7,8]. Misfolded proteins and nutrient excess can trigger the unfolded protein response (UPR) through the actions of three transmembrane ER stress sensor proteins (PERK, IRE1, and ATF6) in an attempt to restore ER homeostasis [9,10]. However, prolonged fatty acid exposure can exceed the capacity to maintain homeostasis, resulting in chronic and unresolved ER stress, especially in the liver, leading to the expression of lipogenic and inflammatory genes and the development of hepatic steatosis [11]. Indeed, studies in humans and rodent models of obesity and NAFLD showed activation of ER stress markers in the liver, indicating that ER stress has a contributing role in the development and progression of NAFLD [11]. Thus, identifying novel UPR regulators capable of relieving hepatic ER stress could reveal potential pharmacological targets for NAFLD.

The mammalian family of NAD^+^-dependent sirtuin deacylases (SIRT1-7) [12] has been implicated in numerous biological functions, including stress response, aging, and metabolism [13]. In recent years, sirtuins have been considered attractive drug targets for metabolic and age-related diseases [14]. The predominantly cytosolic isoform SIRT2 is one of the least understood sirtuins. While its role in cell cycle regulation [15,16], neuroprotection [17,18], and tumor suppression [16,19] has been previously established, other studies suggest that SIRT2 also acts as a key player in various metabolic processes [20]. Interestingly, SIRT2 is downregulated in adipose tissue from obese subjects [21] and in insulin-resistant hepatocytes and livers [22]. Importantly, in our previous study we showed that SIRT2 counteracts insulin resistance in hepatocytes by mitigating oxidative stress and mitochondrial dysfunction [22], which are common pathogenic events in NAFLD. Furthermore, it has been recently demonstrated that SIRT2 prevents liver steatosis by deacetylating the transcription factor hepatocyte nuclear factor 4α (HNF4α) [23]. However, the putative role of SIRT2 in the regulation of hepatic steatosis and the interplay with ER stress has not been previously investigated.

Therefore, the present study aimed to determine the effects of SIRT2 on the regulation of hepatic steatosis in cells and in mice and to explore the underlying signaling pathways.

## 2. Results

### 2.1. SIRT2 Deletion in Mice Aggravates HFD-Induced Obesity

**Figure 1:**
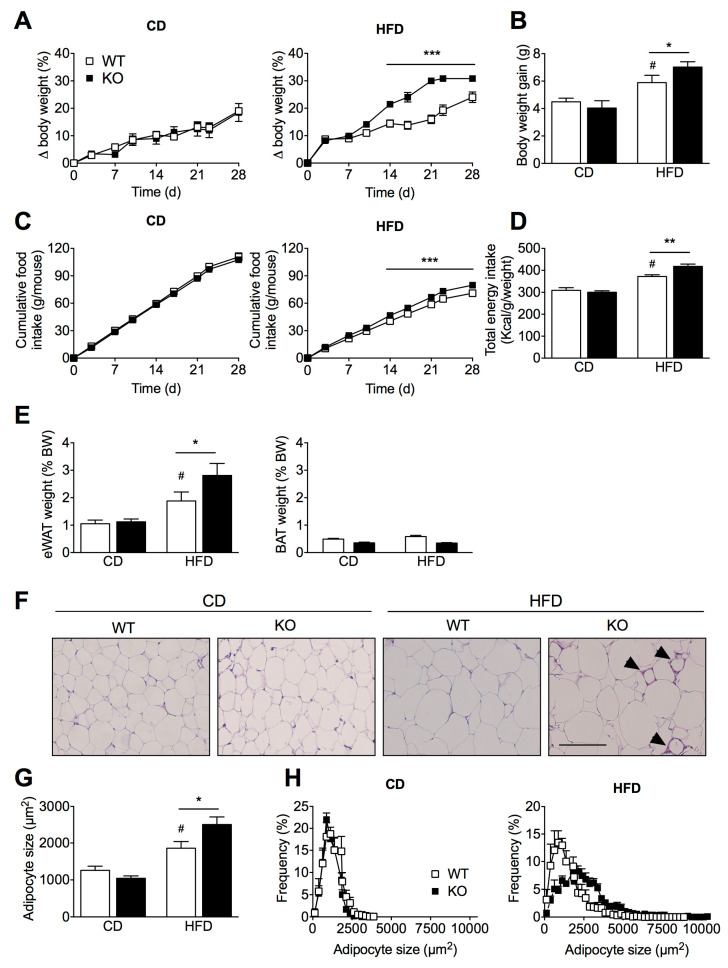
Loss of SIRT2 promotes body weight gain, increased food intake, and adiposity under HFD conditions. (**A**) Body weight gain (%) of WT and SIRT2-KO mice maintained on a CD or a HFD for 4 weeks. (**B**) Total body weight gain (g) at the end of the experiment in WT and SIRT2-KO mice fed a CD or a HFD. (**C**) Cumulative food intake of WT and SIRT2-KO mice on a CD or a HFD. (**D**) Total energy intake (Kcal/weight) calculated at the end of dietary protocol. (**E**) WAT and BAT weight are expressed as a percentage of body weight (BW) of WT and SIRT2-KO mice fed a CD or a HFD. (**F**) Representative images of hematoxylin and eosin (H&E)-stained eWAT sections from WT and SIRT2-KO mice fed a CD or a HFD (scale bar = 100 μm). Arrows indicate “crown-like” structures. (**G**) Adipocyte size and (**H**) frequency distribution using ImageJ software. Data are presented as mean ± SEM; *n* = 5 per group; # *p* < 0.05 compared with CD-fed WT mice; * *p* < 0.05, ** *p* < 0.01, *** *p* < 0.001 compared with HFD-fed WT mice.

To investigate the functional consequences of chronic SIRT2 deletion in vivo, we performed a metabolic analysis of wild-type (WT) and SIRT2-KO mice fed either a chow diet (CD) or a high-fat diet (HFD) for 4 weeks, starting at 8 weeks of age. Genetic deletion of SIRT2 was confirmed by Western blotting analysis (Supplementary Figure S1). When fed a CD, SIRT2-KO mice were similar to WT littermates in terms of body weight gain (Figure 1A,B) and cumulative food intake (Figure 1C,D). However, when challenged with a HFD, SIRT2-KO mice gained substantially more body weight than WT littermates (Figure 1A,B), which was associated with enhanced cumulative food intake (Figure 1C,D). Consistent with increased body weight, the visceral adipose depot (eWAT) was heavier in both genotypes upon HFD, with SIRT2-KO mice showing even higher adiposity (Figure 1E), whereas no differences were found in the brown adipose tissue weight (Figure 1E). Given our observations that SIRT2-KO mice were more susceptible to HFD-induced weight gain and adiposity, we measured changes in adipocyte size in epididymal fat pads. Histological analysis revealed that HFD induced adipocyte hypertrophy in both groups, but this effect was significantly higher in SIRT2-KO mice (Figure 1F,G), which is reflected by a higher frequency of larger adipocytes in SIRT2-KO mice compared to WT mice (Figure 1H). Of note, increased infiltration of macrophages into the fat cells was apparent in HFD-fed SIRT2-KO mice, as evidenced by the crown-like structures surrounding adipocytes in eWAT of SIRT2-KO mice (Figure 1F). In combination, these findings suggest that the loss of SIRT2 aggravates dietary-induced obesity and results in unhealthy expansion of visceral adipose tissue.

### 2.2. SIRT2 Deletion Promotes Glucose Intolerance and Insulin Resistance

**Figure 2:**
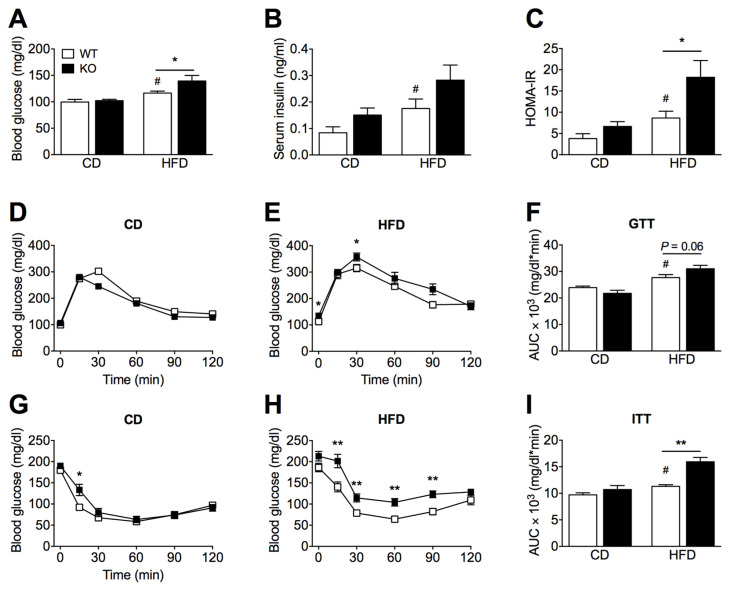
Loss of SIRT2 exacerbates HFD-induced increase in blood glucose levels, glucose intolerance, and insulin resistance. (**A**,**B**) Blood glucose levels (mg/dL) and serum insulin levels (ng/mL) of WT and SIRT2-KO mice fed a CD or a HFD at the end of the experiment measured after 15 h of fasting. (**C**) Insulin resistance score, HOMA-IR, is calculated as fasting plasma glucose times fasting serum insulin divided by 22.5. (**D**,**E**) Intraperitoneal glucose tolerance test (ipGTT) was conducted in WT and SIRT2-KO mice after 24 days of CD or HFD feeding. Blood glucose levels of WT and SIRT2-KO mice at different time points after i.p injection of D-glucose (1.5 g glucose/kg body weight). (**F**) Average AUC of the ipGTT curves was calculated using the trapezoidal rule. (**G**,**H**) Intraperitoneal insulin tolerance test (ipITT) was performed in WT and SIRT2-KO mice fed a CD HFD after 28 days. Blood glucose levels at different time points after i.p injection of human insulin (0.75 U/kg of body weight). (**I**) AUC was calculated using the trapezoidal rule. Data are presented as mean ± SEM; *n* = 7–8 per group; # *p* < 0.05 compared with CD-fed WT mice; * *p* < 0.05, ** *p* < 0.01 compared with HFD-fed WT mice.

We next examined the impact of SIRT2 deficiency on whole-body glucose homeostasis and insulin sensitivity in vivo. Baseline fasting glucose or insulin levels were not significantly different between SIRT2-KO and WT mice on a CD (Figure 2A,B). However, compared to WT mice fed a HFD, SIRT2-KO mice showed an increase in glucose levels (Figure 2A) and a trend for increased insulin levels (Figure 2B). We further evaluated glucose homeostasis by calculating the homeostasis model assessment of insulin resistance (HOMA-IR) and by performing glucose and insulin tolerance tests (GTTs and ITTs). In the GTT, the glucose excursion curve of WT mice fed a HFD was increased compared to CD, while HFD-fed SIRT2-KO mice showed a tendency (*p* = 0.06) for aggravated glucose intolerance (Figure 2D–F). HFD feeding promoted insulin resistance in both genotypes when evaluated by HOMA-IR (Figure 2C) or ITT (Figure 2G–I); however, SIRT2-KO mice were significantly more insulin resistant than WT mice (Figure 2H,I). Thus, SIRT2 deficiency in mice leads to impaired glucose homeostasis and exacerbates insulin resistance under obesogenic conditions.

### 2.3. SIRT2 Deletion Promotes Hepatic Steatosis and Alters the Expression of Key Lipogenic Genes

**Figure 3:**
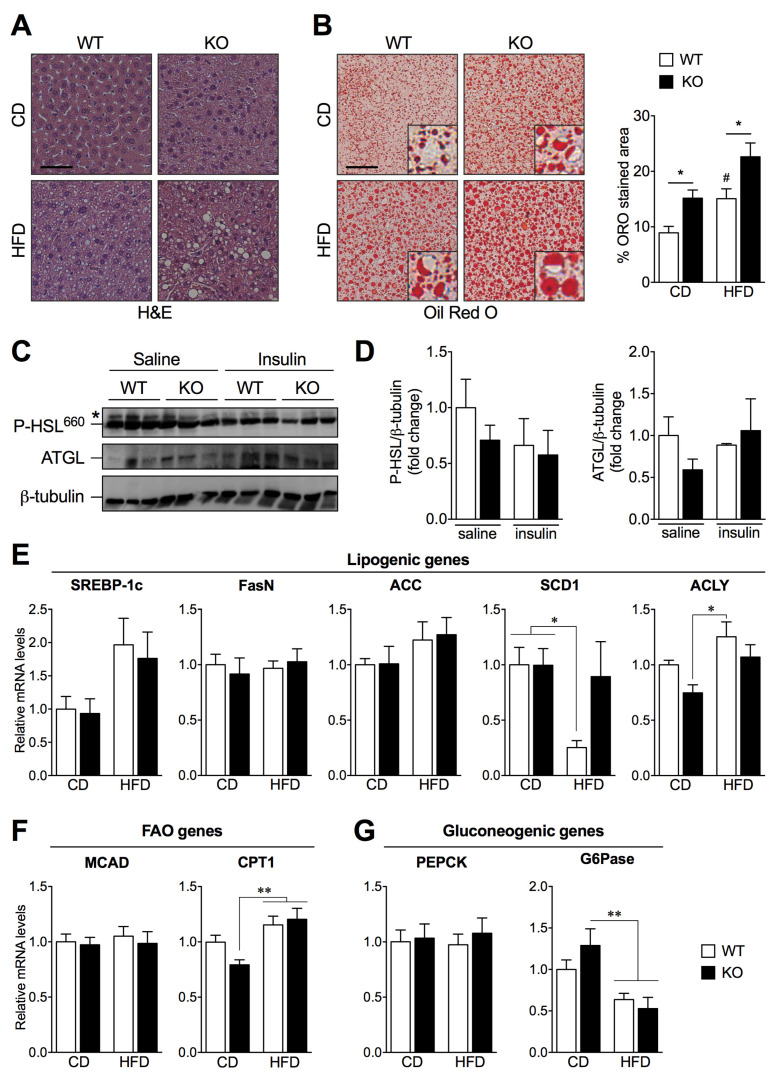
Loss of SIRT2 promotes hepatic steatosis and alters the expression of key genes involved in de novo lipogenesis, fatty acid oxidation, and gluconeogenesis. (**A**) Representative images of H&E-stained and (**B**) ORO-stained liver sections of WT and SIRT2-KO mice fed a CD or a HFD, *n* = 5 per group. ORO stained area (%) was quantified using ImageJ software (scale bar = 50 μm). (**C**) Representative Western blots of adipose tissue lysates (50 μg) showing P-HSL and ATGL expression levels of WT and SIRT2-KO mice fed a HFD, upon saline or insulin stimulation (i.p. injection 2 U/kg). (**D**) Levels of P-HSL and ATGL were normalized to β-tubulin in the same lane. All P-HSL/β-tubulin and ATGL/β-tubulin ratios were normalized to the value from WT mice injected with saline. Each lane corresponds to a distinct animal. *n* = 3 per group. (**E**–**G**) RT-qPCR analysis of lipogenic (SREBP-1c, FasN, ACC, SCD1, and ACLY), fatty acid oxidation (MCAD, CPT1), and gluconeogenic (PEPCK and G6Pase) genes in livers of WT and SIRT2-KO mice fed a CD or a HFD. Results were normalized to reference gene Ywhaz and represented as mean ± SEM; *n* = 5–6 per group; # *p* < 0.05 compared with CD-fed WT mice; * *p* < 0.05, ** *p* < 0.01.

To determine whether SIRT2 has a role in hepatic steatosis, we performed hematoxylin and eosin (H&E) staining of liver sections that revealed normal structural features of hepatocytes from WT and SIRT2-KO mice fed a CD (Figure 3A). Upon HFD, cytoplasmic vacuolization is visible in hepatocytes from WT mice and exacerbated in SIRT2-KO mice (Figure 3A). We next used Oil Red O (ORO) staining to assess hepatic neutral lipid accumulation and found that HFD-fed SIRT2-KO mice show increased lipid accumulation (steatosis) compared to WT mice (Figure 3B). Interestingly, SIRT2-KO mice fed a CD spontaneously exhibit increased hepatic steatosis when compared to WT mice (Figure 3B). Together, these results suggest that the loss of SIRT2 promotes hepatic steatosis, a hallmark of NAFLD, independently of diet.

We next sought to determine the mechanisms underlying increased hepatic steatosis in SIRT2-KO mice fed a HFD. Because impaired adipose tissue lipolysis results in increased flux of free fatty acids into the liver, we investigated the protein levels of major lipolytic enzymes in WAT, namely adipose triglyceride lipase (ATGL) and phosphorylated hormone-sensitive lipase (P-HSL) (Figure 3C,D). ATGL presented no differences between experimental groups; as for P-HSL, no alterations were detected regarding genotype nor upon acute insulin stimulation, suggesting that the loss of SIRT2 does not impact lipolysis. Additionally, serum levels of cholesterol were increased upon HFD in both genotypes, while triglyceride levels were not altered (Supplementary Figure S2). We next examined the mRNA levels of key regulators of lipid and glucose homeostasis in livers from SIRT2-KO and WT mice under HFD feeding. The mRNA levels of the lipogenic genes sterol regulatory element-binding protein-1c (SREBP-1c), fatty acid synthase (FasN), and acetyl-CoA carboxylase (ACC) were not altered in any of the conditions, while stearoyl-CoA desaturase 1 (SCD1) was altered upon HFD in WT mice, and ATP-citrate lyase (ACLY) was increased in WT HFD mice compared to SIRT2-KO mice fed a CD (Figure 3E). Regarding fatty acid oxidation (FAO) gene analysis, carnitine palmitoyl transferase 1 (CPT1) was increased in SIRT2-KO mice fed a HFD compared to SIRT2-KO fed a CD, while medium chain acyl-CoA dehydrogenase (MCAD) was not altered (Figure 3F). Additionally, the gluconeogenic gene phosphoenolpyruvate carboxykinase (PEPCK) was not altered, while glucose-6-phosphatase (G6Pase) expression levels were decreased upon HFD compared to SIRT2-KO mice fed a CD (Figure 3G). The dysregulation of some metabolic genes involved in lipogenesis and FAO in SIRT2-KO together with the increased hepatic lipid accumulation suggest that SIRT2 has a crucial role in regulating hepatic steatosis.

### 2.4. SIRT2 Silencing in HepG2 Cells Increases Lipid Deposition while SIRT2 Overexpression Attenuates Palmitate-Induced Lipid Overload

**Figure 4:**
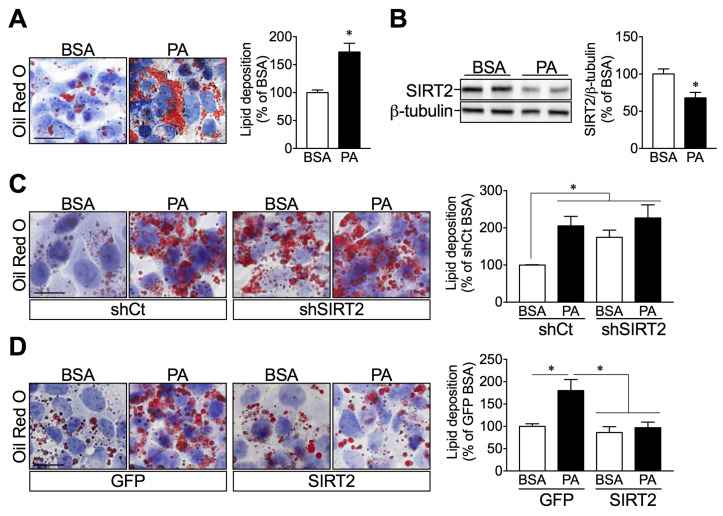
Palmitate and SIRT2 silencing promote lipid accumulation in HepG2 cells, while SIRT2 overexpression attenuates palmitate-mediated lipid deposition. (**A**) HepG2 cells were incubated with BSA (vehicle) or palmitate (0.5 mM) for 24 h. Representative images of ORO-stained HepG2 cells and quantification of ORO-stained lipid droplets by spectrophotometric analysis after isopropanol extraction. (**B**) Representative Western blots of SIRT2. SIRT2 levels were normalized to the levels of β-tubulin in the same lane. (**C**,**D**) Images of ORO-stained HepG2 cells infected with lentiviruses expressing either a short hairpin for control (shCTRL) and SIRT2 (shSIRT2) to silence SIRT2, as well as a vector with GFP sequence (GFP) and SIRT2 sequence (SIRT2) for the overexpression. These cells were incubated with either BSA or palmitate for 24 h and spectrophotometric analysis of stained lipids. (scale bar = 50 μm). Results are presented as mean ± SEM; *n* = 3 per group; * *p* < 0.05.

We further studied if the increased hepatic lipid accumulation in SIRT2-KO mice resulted from the loss of SIRT2 expression. To test this hypothesis, we used an established in-vitro model of hepatic steatosis by treating human hepatic HepG2 cells with palmitate. As expected, palmitate induced intracellular lipid accumulation, as assessed by Oil Red O staining, using both microscopic analysis and spectrophotometric quantification (Figure 4A), which was accompanied by a significant decrease in SIRT2 protein expression levels (Figure 4B). Next, to determine a causal relationship between SIRT2 expression and hepatic lipid accumulation, we used a genetic approach with lentiviral vectors to either silence or overexpress SIRT2. Both silencing and overexpression of SIRT2 were confirmed by Western blotting (Supplementary Figure S3). Importantly, SIRT2-silenced cells showed increased lipid droplets similar to what occurred when control cells were treated with palmitate (Figure 4C). Conversely, SIRT2 overexpression in hepatocytes prevented palmitate-induced intracellular lipid accumulation (Figure 4D). These results further support a protective role of SIRT2 in maintaining hepatic lipid homeostasis under lipid overload conditions.

### 2.5. SIRT2 Links ER Stress and Hepatic Lipid Accumulation

**Figure 5:**
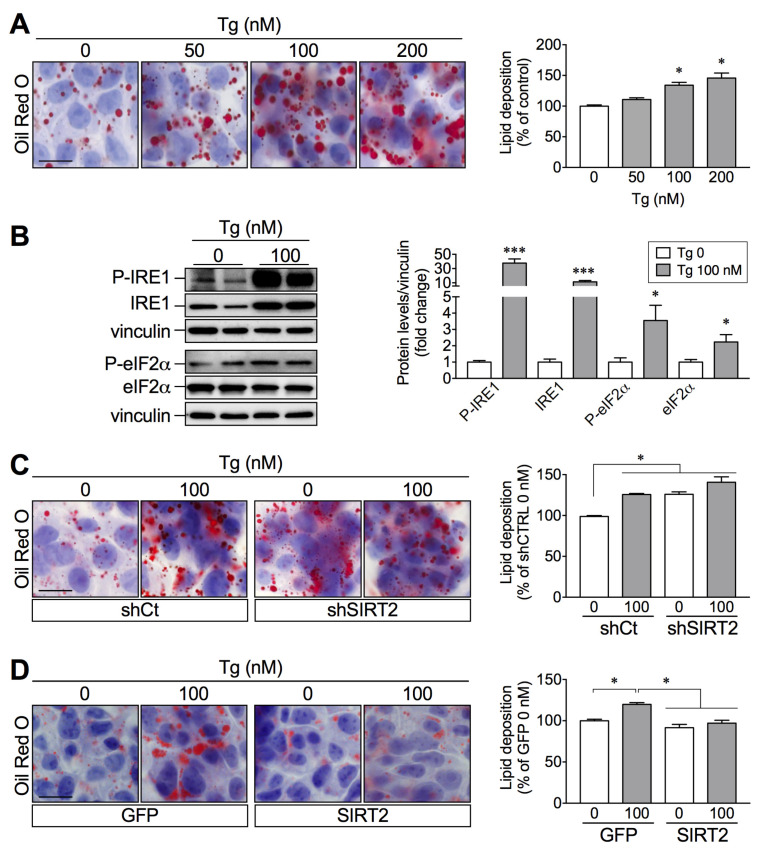
Thapsigargin-induced lipid accumulation is prevented by SIRT2 overexpression. (**A**) Representative images of ORO-stained HepG2 cells incubated with increasing thapsigargin (Tg) concentrations (0, 50, 100, 200 nM) for 24 h and spectrophotometric quantification of intracellular lipids. (**B**) Representative Western blots of P-IRE1 (Ser724), IRE1, P-eIF2α (Ser51), and total eIF2α of HepG2 cells treated with vehicle or Tg (100 nM) for 24 h. All target protein levels were normalized to the levels of the housekeeping protein in the same lane. Values in the graph were normalized for the vehicle control (0 Tg). (**C**,**D**) Representative images and spectrophotometric quantification of intracellular lipids of ORO-stained HepG2 cells infected with lentiviruses expressing either a short hairpin for control (shCTRL) and SIRT2 (shSIRT2) to silence SIRT2, as well as a vector with GFP sequence (GFP) and SIRT2 sequence (SIRT2) for the overexpression. These cells were treated as in B (scale bar = 50 μm). Results are presented as mean SEM; *n* = 3 per group; * *p* < 0.05, *** *p* < 0.001.

Unresolved ER stress is associated with hepatocyte dysfunction and contributes to the onset and progression of NAFLD [11]. We set to study the link between SIRT2 and ER stress in hepatocytes to further explain the role of SIRT2 in NAFLD. HepG2 cells treated with thapsigargin (Tg), an ER stress inducer, showed a concentration-dependent increase in lipid deposition (Figure 5A), suggesting that ER stress activation may directly contribute to lipid accumulation in hepatocytes. As expected, Tg (100 nM) induced a robust activation of ER stress, as demonstrated by the increased expression of the ER stress markers inositol-requiring enzyme 1-α (IRE1) and eukaryotic initiation factor 2-α (eIF2α), in their phosphorylated and total forms (Figure 5B). To assess whether the effects of SIRT2 on lipid accumulation are mediated through the ER stress response, SIRT2-silenced or SIRT2-overexpressing hepatic cells were incubated with Tg (100 nM). SIRT2 silencing increased lipid accumulation to the same extent as Tg treatment (Figure 5C); conversely, SIRT2 overexpression abrogated Tg-induced lipid accumulation (Figure 5D). Together, these results propose a link between SIRT2 levels and ER stress activation in the development of lipid accumulation, a hallmark of NAFLD.

### 2.6. SIRT2 Overexpression in HepG2 Cells Attenuates Palmitate-Induced ER Stress Activation

**Figure 6:**
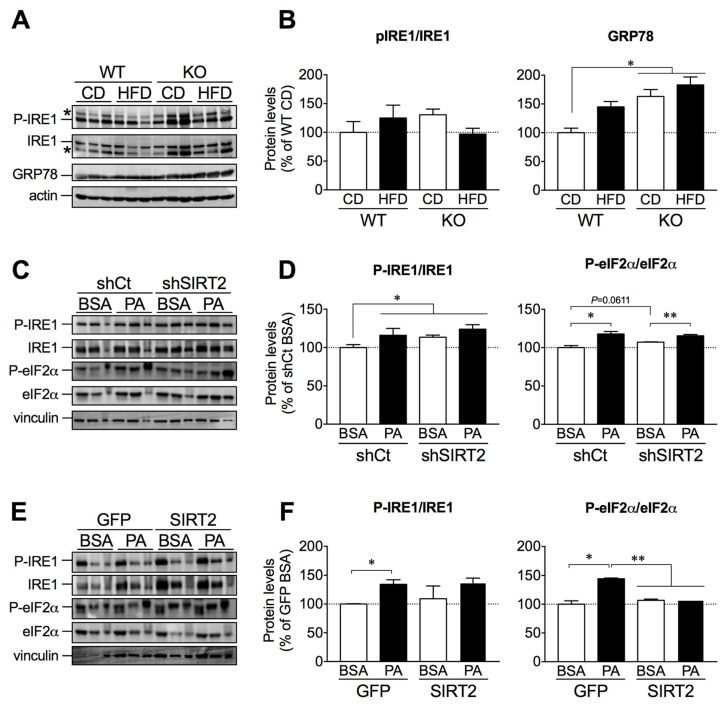
SIRT2 overexpression blunted palmitate-induced ER stress activation. (**A**) Representative Western blots of liver lysates showing P-IRE1 (Ser724), IRE1, and GRP78 levels from WT and SIRT2-KO mice fed a CD or HFD. (**B**) In the same lane, levels of P-IRE1 (Ser724) were normalized to IRE1, while GRP78 levels were normalized to actin. Values in the graph were normalized for the control condition (WT CD). *n* = 5–6 per group. (**C**) Representative Western blots of P-IRE1 (Ser724), IRE1, P-eIF2α (Ser51), and eIF2α of control HepG2 cells or SIRT2-silenced cells treated with BSA (vehicle) or palmitate (0.5 mM) for 24 h. (**D**) In the same lane, levels of P-IRE1 (Ser724) and P-eIF2α (Ser51) were normalized to IRE1 and eIF2α, respectively. Values in the graph were normalized for the control (shCT BSA). *n* = 3 per group. (**E**) Representative Western blots of P-IRE1 (Ser724), IRE1, P-eIF2α (Ser51), and eIF2α of control HepG2 cells or SIRT2-overexpressing cells treated with BSA (vehicle) or palmitate (0.5 mM) for 24 h. (**F**) In the same lane, levels of P-IRE1 (Ser724) and P-eIF2α (Ser51) were normalized to IRE1 and eIF2α, respectively. Values in the graph were normalized for the control (BSA GFP). *n* = 3 per group; * *p* < 0.05, ** *p* < 0.01.

To further establish the link between lipid accumulation, ER stress activation, and SIRT2 levels, we assessed protein expression levels of P-IRE1, IRE1, and glucose related protein-78 (GRP78) in livers from WT and SIRT2-KO mice fed a CD or HFD. Although we observed no significant changes in P-IRE1/IRE1 expression, GRP78 expression was increased in SIRT2-KO mice fed a CD or HFD (Figure 6A,B). We next assessed ER stress markers in SIRT2-silenced or SIRT2-overexpressing cells treated with vehicle (BSA) or palmitate. As expected, palmitate induced ER stress activation in control cells (Figure 6D,F). In agreement with the results on lipid accumulation, SIRT2 silencing led to increased expression of ER stress proteins (Figure 6C,D). In contrast, SIRT2 overexpression prevented palmitate-induced P-eIF2α expression (Figure 6E,F). These results indicate that SIRT2 exerts a protective role against palmitate-induced ER stress activation.

## 3. Discussion

In this study, we investigated the contribution of SIRT2 to the regulation of metabolic homeostasis. Our data suggest that SIRT2 regulates glucose homeostasis and hepatic lipid accumulation, potentially through the ER stress response. We found that SIRT2 deficiency in mice exacerbates HFD-induced obesity, glucose intolerance, and insulin resistance. Moreover, SIRT2 ablation leads to hepatic steatosis independently of diet and increased ER stress response. In-vitro studies indicate that both SIRT2 silencing and chemical induction of ER stress enhance intracellular lipid accumulation, while SIRT2 overexpression prevents both fatty acid- and ER stress-induced lipid accumulation. Together, these observations highlight the protective role of SIRT2 in conditions of lipid overload and suggest that SIRT2 activation may improve the development of NAFLD by impacting mechanisms of lipid accumulation and ER stress activation.

Our work and many previous studies have used genetic models combined with dietary protocols to study molecular mechanisms involved in metabolic health and disease. While it may not allow a direct extrapolation to a clinical setting, it will help clarify the function of molecular targets in biological processes with clinical relevance [18,24]. Research on NAFLD/NASH focuses on identifying therapeutic targets to improve or treat this condition, although weight loss remains the most effective approach to managing the disease. Sirtuins are promising targets in the treatment of metabolic diseases. SIRT1 activation by resveratrol, particularly, has been largely explored. A clinical trial in overweight and obese insulin-resistant subjects reported no effect of resveratrol on liver fat content after 12 weeks of treatment [25]. In contrast, a study in patients with NAFLD showed a protective effect of resveratrol after 6 months of treatment [26]. While preclinical drug research for NAFLD/NASH using genetically engineered rodents and dietary interventions may not have identified any effective drug to date, the study of molecular mechanisms and pharmacological compounds must continue as it may prove successful, similar to what happened in the past with other diseases.

In our work, WT and SIRT2-KO mice fed a CD exhibit similar body weight gain, food intake, glucose tolerance, and insulin sensitivity, suggesting that SIRT2 is likely to be dispensable for metabolic homeostasis under regular diet conditions. In contrast, a previous report showed increased body weight and insulin resistance in CD-fed SIRT2-KO mice with no alterations in food intake or energy expenditure, but with increased lean and fat mass, suggesting this explanation for the increased body weight [24]. The impact of dietary fat on the development of features of the metabolic syndrome can be observed as early as 3 days, with studies in mice showing increased body weight, food intake, glucose and insulin resistance, and increased lipid content in the liver and skeletal muscle [27]. Here, upon 4 weeks of HFD feeding, SIRT2-KO mice have increased body weight gain and adiposity, which may be due to increased food intake in SIRT2-KO mice. Similar to our findings, a previous report showed increased body weight in HFD-fed SIRT2-KO mice compared to WT mice, accompanied by increased food intake, despite higher energy expenditure during the dark cycle [24]. The authors suggested a possible role for SIRT2 in regulating satiety and food intake of highly palatable foods at the central level [24]. Our data support the role of SIRT2 in the regulation of body weight, as shown by the expansion of adipose tissue mass in HFD-fed SIRT2-KO mice, with increased adipocyte size and decreased adipocyte number. The finding that SIRT2 deficiency leads to increased adipogenesis is consistent with previous studies showing that SIRT2 regulates adipocyte differentiation [28,29] and thus adipose tissue mass and function. Furthermore, adipose tissue expansion can also lead to lipotoxicity, the abnormal accumulation of lipids in peripheral non-adipose tissues, and this process is related to lipolysis impairment. More recently, lipophagy—a selective form of autophagy that degrades lipid droplets—has also been associated with the development and progression of NAFLD, and activating this pathway has been shown to ameliorate NAFLD in a SIRT1-dependent manner [30]. In the eWAT, the lipolysis regulators ATGL and HSL were not altered with HFD or with the loss of SIRT2. However, SIRT2 has been associated with increased lipolysis through the deacetylation of FOXO1 and regulation of PPARγ, as observed in an adipocyte cell line [29]. Our finding that SIRT2-KO mice were markedly insulin resistant upon HFD is in line with our prior observation that SIRT2 is involved in the regulation of insulin sensitivity [22] and other reports showing the interaction between SIRT2 and Akt [31]. We observed a decrease in G6Pase gene expression in mice fed a HFD, while others reported increased G6Pase and PEPCK gene expression in mice with hepatic SIRT2 deficiency, resulting in reduced gluconeogenesis, which was reversed by hepatic SIRT2 overexpression [23].

Evidence of an association between fatty liver and sirtuins has accumulated in recent years. It was reported that SIRT1, SIRT3, SIRT5, and SIRT6 expression are decreased in the livers of NAFLD patients [32]. In mouse studies, ablation of SIRT1 in the liver [33] or global SIRT7 deletion [34] led to fatty liver under standard diet conditions, and global SIRT3 ablation accelerated steatosis development under HFD [35].

Highlighting the importance of SIRT2 on the homeostasis of liver function, a very recent study showed that hepatic SIRT2 expression is decreased in NAFLD patients. Moreover, hepatocyte-specific SIRT2 deficiency in mice aggravates HFD-induced insulin resistance, glucose intolerance, and hepatic steatosis and associated inflammatory markers. These phenotypes were opposed by overexpressing SIRT2 in the liver [23]. Accordingly, our study shows that SIRT2 ablation is sufficient to induce steatosis on a regular diet, which was exacerbated with the consumption of a HFD. Hepatic lipid accumulation may result from increased de-novo lipogenesis, a process primarily regulated by the transcription factor SREBP-1c, reported to be increased upon HFD. Our data did not show a significant alteration of this gene upon HFD or differences between genotypes, nor its downstream gene, SCD1. However, it has been shown that SREBP gene expression may be altered depending on feeding or fasting state, where fasting decreases SREBP-1c levels [36], which may explain the lack of alteration in the liver of these animals. Similarly, while most reports show an increase in SCD1 expression under NAFLD conditions, a decrease has also been observed [37]. In this work, we did not show SIRT2 interaction with SREBP-1c; however, it has been shown that SIRT2 regulates SREBP-2 of the same family. In neuronal cells, SIRT2 inhibition led to the decrease of cholesterol synthesis, thus supporting the importance of SIRT2 in the maintenance of homeostasis through the regulation of lipid metabolism [18].

ER stress activation has already been linked to the development and progression of NAFLD. In this study, we show a link between SIRT2 deficiency and ER stress activation, demonstrated by the increased expression of the ER stress marker GRP78 in SIRT2-KO mice, independent of diet. We further set to study the connection between SIRT2 and ER stress in lipid overload conditions. ER stress activation was observed in HepG2 cells treated with palmitate due to lipid overload [38]. Furthermore, mice subjected to pharmacological ER stress activation developed fatty liver [39,40]. Herein, the chemical ER stress inducer thapsigargin triggered lipid accumulation in HepG2 cells, while SIRT2 overexpression counteracted this effect. Likewise, SIRT2 overexpression blunted the expression of proteins associated with the activation of ER stress upon incubation with palmitate. The data indicate a possible role for SIRT2 in the ER stress response pathway. In this regard, the natural compound silibin exerted hepatoprotective activity in mice with NAFLD by inhibiting ER stress and NRPL3 inflammasome activation through a SIRT2-dependent pathway [41].

Considering the functional redundancies and overlapping actions of sirtuin members [13], it is plausible that the mechanisms by which SIRT2 modulates the ER stress response may be similar to those of other sirtuins. SIRT7 modulated ER stress activation and prevented hepatic steatosis by inhibiting the transcription factor Myc [34]. In the case of SIRT3, reduced deacetylase activity in livers of HFD-fed mice leads to hyperacetylation, among others, of the GRP78 protein of the ER stress pathway [42]. Additionally, loss of SIRT6 induced hepatic steatosis through the deacetylation of X-box-binding protein 1 (XBP1s), a key modulator of ER stress, while SIRT6 overexpression prevented ER stress activation triggered by tunicamycin, a well-known ER stress inducer [43]. Also, SIRT1 activation by caloric restriction and resveratrol reduced lipid accumulation with a concomitant decrease of ER stress markers [44]. Although the beneficial effects of caloric restriction are largely attributed to SIRT1 activation, resveratrol can modulate the activity of other sirtuins [45], making it possible that SIRT2 activation may improve steatosis through an ER stress-dependent pathway [44].

There remain several limitations of our study, including a more in depth analysis of the mechanisms underlying the regulation of ER stress by SIRT2. Future in-vitro and in-vivo studies involving the manipulation of ER stress with tauroursodeoxycholic acid (TUDCA), a chemical chaperone that relieves ER stress, are required to clarify these processes and further consolidate the role of SIRT2 in ER stress regulation.

Overall, this work highlights the relevance of SIRT2, particularly in the context of HFD-induced metabolic dysfunction, where its deficiency leads to disruption of hepatic lipid homeostasis and the development of NAFLD. It further shows that enhancing SIRT2 expression protects against lipid overload induced by palmitate, a saturated fatty acid highly abundant in the Western diet, and underscores the potential role of SIRT2 in ER stress modulation. SIRT2 activation might constitute a therapeutic strategy against obesity and its metabolic complications.

## 4. Materials and Methods

### 4.1. Animals and Diets

Seven-week-old male C57BL/6 wild-type (WT) mice and B6.129-Sirt2^tm1.1Fwa^ (SIRT2-KO) mice were acquired from Charles River Laboratories (Barcelona, Spain) and The Jackson Laboratory (Bar Harbor, ME, USA), respectively. SIRT2-KO mice were generated in B6.129 genetic background and backcrossed to C57BL/6 for at least 8 generations to produce homozygous SIRT2^−/−^ mice [16]. A colony of SIRT2-KO mice was established at the Centre for Neuroscience and Cell Biology (CNC), University of Coimbra, Portugal. Mice (*n* = 4 per cage) were maintained in a temperature-controlled facility (23 °C) with a 12 h light/dark cycle and given free access to standard chow diet and water. Mice were allowed to adapt to the laboratory conditions for at least 1 week before the onset of the dietary protocol. Subsequently, mice were fed either a standard chow diet (CD, 4RF21, Mucedola, Milan, Italy) or a high-fat diet (HFD, 60% kcal from fat, D12492, Research Diets, New Brunswick, NJ, USA) for 4 weeks (for details on diet composition, see Supplementary Table S1). Food intake per cage and individual body weights of mice were recorded twice per week. Food intake per mouse was calculated by dividing food intake by the number of mice in the cage and adjusted for body weight gain over a specific time period. Experimental procedures were approved by the CNC animal experimentation board (ORBEA 165) and were performed following the European Community directive (86/609/EEC).

### 4.2. Glucose and Insulin Tolerance Tests

Intraperitoneal (i.p.) glucose tolerance tests (GTT) and insulin tolerance tests were conducted after 24 and 28 days of dietary intervention, respectively. To measure glucose tolerance, mice were fasted overnight (~15 h) and glucose (1.5 g/kg body weight) was administered by i.p. injection of a 20% glucose solution in 0.9% saline. Tail blood glucose was measured at 0 (basal), 15, 30, 60, 90, and 120 min after injection using a glucometer (FreeStyle Precision Neo, Abbott, Chicago, IL, USA). To measure insulin sensitivity, mice were fasted for 6 h and then i.p. injected with insulin (0.75 U/kg body weight, Humalog, Lilly). Tail blood glucose was measured at 0, 15, 30, 60, 90, and 120 min as for the GTT. The area under the curve (AUC) of the GTT and ITT was calculated by the trapezoidal rule.

### 4.3. Plasma Biochemistry

Mice were sacrificed after overnight fasting with a lethal dose of halothane followed by decapitation. Liver and epididymal white adipose tissue (eWAT) were collected, weighed, and immediately snap frozen in liquid nitrogen and stored at −80 °C or fixed in formaldehyde (10%) and paraffin-embedded. Trunk blood was collected and serum was separated by centrifugation (2000× *g*, 15 min) and stored at −20 °C. Blood glucose concentrations from tail blood were measured with a glucometer and glucose strips. Serum insulin levels were measured with a commercially available ELISA kit (EMD Millipore, Burlington, MA, USA), according to manufacturers’ instructions.

### 4.4. Tissue Histology

Paraffin-embedded liver and eWAT blocks were sectioned (4 μm) on a microtome (Thermo Scientific HM325, Waltham, MA, USA) and stained with hematoxylin and eosin (H&E) using standard procedures. Frozen liver blocks were cut into 8–10 μm-thick sections on a cryostat-microtome (Leica CM3050S, Allendale, NJ, USA) for Oil Red O (ORO) staining to visualize neutral lipids. ORO stained area (%) was quantified using NIH ImageJ software (see online Supplementary Materials and Methods for details).

### 4.5. Cell Culture and Treatments

HepG2 cells, a human hepatocyte-derived cell line, were obtained from ATCC (Rockville, MD, USA). Cells were cultured at 37 °C in a humidified atmosphere with 5% CO_2_ in Minimum Essential Medium (MEM) containing 1 g/L glucose, 10% heat-inactivated fetal bovine serum (FBS), and 1% penicillin/streptomycin. Cells were cultured in 75 cm^2^ flasks (Thermo Fisher Scientific, Waltham, MA, USA) and plated in either 6 or 12 multi-well plates (Thermo Fisher Scientific, Waltham, MA, USA), at a density of 1 × 10^6^ or a 5 × 10^5^ cells/well, respectively. When reaching 70–80% confluency, cells were subcultured using Trypsin-EDTA solution. To induce lipid deposition, cells were treated for 24 h with 0.5 mM palmitate (Sigma, St. Louis, MO, USA) coupled to fatty acid-free bovine serum albumin (BSA) (Calbiochem, San Diego, CA, USA) in serum-free MEM, as an established in-vitro model of steatosis. Uncoupled BSA (1%) was used in control conditions. ER stress was induced by incubating the cells with thapsigargin (Tg) for 24 h. Dimethylsulfoxide (DMSO) alone was used as a control treatment. Cell viability was assessed by the Alamar Blue assay to determine the toxicity of the compounds employed. No significant differences in cell viability were observed in HepG2 cells exposed to any of these compounds (data not shown).

### 4.6. ORO Staining of HepG2 Cells

Lipid accumulation in HepG2 cells was determined by Oil Red-O staining (Sigma, St. Louis, MO, USA). Cells grown on 12-well plates were fixed with 4% paraformaldehyde and stained with ORO (0.625% in 100% isopropanol). Excess dye was removed, and images were acquired using a brightfield microscope (Zeiss, Jena, Germany). Intracellular lipid content was obtained by extracting the ORO dye from the stained cells using 100% isopropanol and quantified spectrophotometrically at 520 nm [46]. Absorbance results are presented as a percentage of the control group.

### 4.7. Production of Lentiviral Vectors

Lentiviral vectors encoding SIRT2 (LV-PGK-SIRT2) for the overexpression, the control GFP (LV-PGK-GFP), silencing shRNA targeting SIRT2 (LV-PGK-EGFP-H1-shSIRT2), and control (LV-PGK-EGFP-H1-shCTRL) were produced in the HEK293T cell line with a four-plasmid system, as previously described [47]. Lentiviral particles were resuspended in 1% BSA in sterile PBS. The viral particle content of batches was evaluated by assessing HIV-1 p24 antigen levels by ELISA (Retro Tek, Gentaur, Paris, France). Concentrated viral stocks were stored at −80 °C until use.

### 4.8. Generation of SIRT2-Silenced and SIRT2-Overexpressing Stable Transfectants

Lentiviral transduction of HepG2 cells was performed as previously described [48]. For knockdown studies, HepG2 cells were transduced with either a lentivirus expressing shSIRT2 or a scrambled control (shCTRL). To achieve SIRT2 constitutive overexpression, HepG2 cells were transduced with lentiviral vectors containing either SIRT2 (overexpression) or GFP (overexpression control).

### 4.9. Western Blotting Analysis

Western blotting was performed as described in the Supplementary Materials and Methods.

### 4.10. Quantitative Real-Time PCR

Total RNA extraction, reverse transcription, and quantitative real-time PCR (qPCR) analysis were performed as described in the Supplementary Materials and Methods. The sequences of the primers are provided in Supplementary Table S2.

### 4.11. Statistical Analysis

Statistical analysis was conducted using GraphPad Prism software version 7.0 (GraphPad Software Inc., San Diego, CA, USA). Data are expressed as mean ± SEM, representing at least three independent in-vitro experiments or at least two independent in vivo experiments. The numbers of mice used in each experimental setting and analysis are specified in each figure legend. Statistical significance was determined by *t*-Student (when comparing two groups) or ANOVA (when comparing more than two groups). Across all analyses, a *p* value of less than 0.05 was considered significant.

## Data Availability

All data presented in this study are available in the main body of the manuscript.

## Supplementary Materials

The following supporting information can be downloaded at: www.mdpi.com/article/10.3390/ijms23126790/s1.
