# Supplementary material for: SIRT2 Deficiency Exacerbates Hepatic Steatosis via a Putative Role of the ER Stress Pathway
Source: Int J Mol Sci. 2022 Jun 17;23(12):6790. doi: 10.3390/ijms23126790 (PMC9223775; doi:10.3390/ijms23126790)
Supplement: Supplementary file 1 [file ijms-23-06790-s001.zip › ijms-1707054-supplementary/ijms-1707054-supplementary/ijms-1707054-SM.pdf]

SIRT2 deficiency exacerbates hepatic steatosis via a putative role of the ER stress pathway

Helena Leal[1,2,3*], João Cardoso[1,2*], Patrícia Valério[1,2*], Marta Quatorze[1,2], Vítor Carmona[1,2,3], Janete Cunha-Santos[1,2,3], Luís Pereira de Almeida[1,2,3], Cláudia Pereira[1,2,4], Cláudia Cavadas[1,2,3,#], Pedro Gomes[1,2,4,5,#]

[1]CNC - Center for Neuroscience and Cell Biology, University of Coimbra, Portugal; [2]Center for Innovative Biomedicine and Biotechnology (CIBB), University of Coimbra, Portugal; [3]Faculty of Pharmacy, University of Coimbra, Portugal; [4]Faculty of Medicine, University of Coimbra, Portugal; [5]Department of Biomedicine, Faculty of Medicine, University of Porto, Portugal
* equal contribution; # these authors share senior authorship

Corresponding authors:
C. Cavadas (ccavadas@ci.uc.pt); P. Gomes (barrosgomes@uc.pt)
Center for Neuroscience and Cell Biology (CNC), University of Coimbra, Rua Larga, 3004-517 Coimbra, Portugal, 239 820 190

Contents

1. Supplementary Materials and Methods
2. Supplementary Figures

1. Supplementary Materials and Methods

Diets

Table S1: Detailed diet composition.

	**High Fat Diet (D12492)**	**Chow Diet (4RF25)**
**Nutrient information**	Kcal %	Kcal %
Protein	20.0	22.0
Carbohydrate	20.0	3.5
Fat	60.0	50.5
Kcal/g	5.24	2.80
Formula	g/Kg	Representative ingredients
Casein	200	Wheat, Maize Soybean meal extracted toasted, Corn gluten feed, Wheat straw, Fish meal, Lucerne meal, Mineral dicalcium phosphate, Calcium carbonate, Sodium chloride, Whey powder, Soybean oil, Yeasts, Choline chloride, D/L-Methionine, Vitamins
L-Cystein	3	
Corn Starch	0	
Maltodextrin 10	125	
Sucrose	68.8	
Cellulose, BW200	50	
Soybean Oil	25	
Lard	245	
Mineral Mix S10026	10	
DiCalcium Phosphate	13	
Calcium Carbonate	5.5	
Potassium Citrate, 1 H_2O	16.5	
Vitamin Mic V10001	10	
Choline Bitartrate	2	
FD&C Blue Dye #1	0.05	

Hematoxylin and eosin staining

For histological analysis of paraffin sections, epididymal white adipose tissue (eWAT) and liver were stained with hematoxylin and eosin (H&E). Slides were kept for 30 min at 68 °C to melt the paraffin. After two washes in xylene for 3 and 2 min, slides were transferred to a glass coplin jar containing 100% ethanol (v/v) for 4 min and 95% ethanol (v/v) for 2 min and rinsed two times with distilled water for 30 s. Slides were stained in hematoxylin Gill III (Merck KGaA, EMO Millipore Corporation) for 5 min and bathed two times in distilled water for 2 and 1 min. After that, slides were stained with eosin Y solution 0.5% aqueous (Merck KGaA, EMO Millipore Corporation) for 1 min and dehydrated with two fast rinses in water, 95% ethanol (v/v), and 100% ethanol (v/v) in water for 1 min, and two times with xylene for 2 min. Glass slides were then mounted using Richard-Allan Scientific Mounting Medium (Thermo Fisher Scientific).

Oil Red O staining of liver tissue

To analyze hepatic lipid content, ORO staining was performed on frozen liver sections. Livers were cut and collected to microscope slides (SuperFrost, Thermo Scientific). After 10 min

at room temperature, 1 mL ORO working solution (1.5 parts ORO stock solution (1.25 g ORO (Sigma-Aldrich) + 200 mL isopropyl 99% solution) was added to one part of water for 10 minutes, and then rinsed with water to remove the solution. Tissue sections were left to dry and mounted using Mowiol. Microscope images were acquired using Axio Imager Z2 (Transmission Light, Objective plan-Apochromat 20x/0.8 M27, 0,512 µm/pixel). ORO stained area (%) was determined by analyzing 10 images for each animal quantified using ImageJ software.

Adipocyte size measurements

H&E-stained eWAT sections were visualized using Axio Imager Z2 (Transmission Light, Objective plan-Apochromat 20x/0.8 M27, 0,512 µm/pixel) for qualitative and quantitative analysis. Adipocyte measurements (average adipocyte area and diameter) were obtained from at least 200 adipocytes per mice (three slides per mice, four mice *per* group) and quantified using ImageJ software.

Western blotting analysis

Tissues (liver and eWAT) or HepG2 cells were homogenized in ice-cold RIPA buffer containing 50 mM Tris-HCl pH 7.4, 150 mM NaCl, 5 mM EDTA, 1% (v/v) Triton X-100, 0.5% (w/v) sodium deoxycholate, and 0.1% (w/v) sodium dodecyl sulphate (SDS), and supplemented with protease and phosphatase inhibitors [200 µM phenyl-methylsulphonylfluoride (PMSF), 1 mM dithiothreitol (DTT), 1 mM Na_3VO_4; 10 mM NaF and complete mini protease inhibitor cocktail tablet (Roche)]. Lysates were incubated for 30 min on ice and centrifuged for 15 min at 14,000 rpm at 4 °C. The protein concentration of each sample was determined by the bicinchoninic acid (BCA) protein assay (Pierce Biotechnology). Samples were denatured with 6x sample buffer (0.5 M Tris-HCl, 30% glycerol, 10.4% SDS, 0.6 M DTT, 0.02% bromophenol blue; pH 6.8) for 5 min at 95 °C. Equal amounts of total protein (30-50 µg) were separated by 10% SDS-PAGE. Electrophoresis was performed with Tris–Bicine running buffer (25 mM Tris, 25 mM Bicine, 1% SDS (w/v); pH = 8.3) at 70 volts for 10 minutes and 120-130 volts for 70 to 90 minutes. Proteins were transferred to PVDF membranes using CAPS buffer (10 mM CAPS, pH= 11.0 with 10% methanol (v/v)) at 4 °C for 2 hours at 1000 mA. Thereafter, membranes were incubated at room temperature in Blocking Buffer (5% (w/v) BSA diluted in TBS-T (Tris-Buffered Saline Tween; 20 mM Tris-base, 0.137 M NaCl and 0.01% Tween). Membranes were incubated overnight at 4º C with primary antibodies. The following primary antibodies were used: anti-SIRT2 (1:1000; Sigma S8447), polyclonal anti-P-HSL Ser660 (1:1000; Cell Signaling 4126S), monoclonal anti-ATGL (1:1000; Santa Cruz Biotechnology F-7), anti-GFP (Santa Cruz), anti-P-IRE1 Ser724 (1:1000; Abcam S724), anti-IRE (1:1000; Cell Signaling 14C10), anti-P-eIF2a Ser51 (1:1000; Cell Signaling D968), anti-eIF2a (1:1000; Cell Signaling D7D3), anti-GRP78 (1:1000; BD Transduction Laboratories), anti-tubulin (1:10000; Sigma T6074), anti-actin (1:10000; Sigma), and anti-vinculin (1:5000; Millipore MAB3574). Subsequently, membranes were incubated with the corresponding alkaline phosphatase-linked secondary goat anti-mouse or anti-rabbit antibody (1:10000; Thermo Scientific) or horseradish peroxidase-linked secondary anti-mouse or anti-rabbit antibody (1:10000; Cytiva). Bands were visualized using ECF substrate or ECL substrate in the Versa-Doc 3000 imaging system (Bio-Rad, USA). Densitometry of the bands was quantified using NIH ImageJ software.

qPCR analysis

Total RNA was extracted from the liver using the NucleoSpin RNA Isolation kit for tissue (MACHEREY-NAGEL) according to the manufacturer's manual. DNase digestion was performed during the process to exclude any contamination with genomic DNA. Total RNA was quantified by optical density (OD) measurements using a ND-1000 Nanodrop Spectrophotometer (Thermo Scientific), and the purity was assessed with the ratio of OD at 260 and 280 nm. Total RNA samples were stored at -80ºC until they were analyzed. The mRNA levels of genes involved in selected metabolic pathways were assessed by quantitative RT-PCR (qRT-PCR). cDNA was obtained from the conversion of 1000 ng total RNA using the iScript

cDNA Synthesis Kit (Bio-Rad) according to the manufacturer's instructions. For mRNA quantification, the SsoAdvanced SYBR Green Supermix (BioRad) was used in combination with pre-designed PCR primer sets (Invitrogen) (Table S2). The reference gene Ywhaz was validated by GeNorm and NormFinder softwares, and PCR primer sets by PrimerBlast from PubMed and Oligocalc.

Table S2: Primer sequences and annealing temperatures for the genes analyzed by qRT-PCR in the mouse liver.

Gene	Primer Sequence	Annealing T (°C)
G6Pase	F: CTGTGAGACCGGACCAGGA<br>R: GACCATAACATAGTATACACCTGCTGC	60
PEPCK	F: CCAACGTGGCCGAGACTAGCG<br>R: GGCACATGGTTCCGCGTCCT	60
SREBP-1c	F: GATCAAAGAGGAGCCAGTGC<br>R: TAGATGGTGGCTGCTGAGTG	64
ACC	F: GGAGATGTACGCTGACCGAGAA<br>R: ACCCGACGCATGGTTTTCA	56
FasN	F: AGCTTCGGCTGCTGTTGGAAGT<br>R: TCGGATGCCTCTGAACCACTCACA	60
SCD1	F: CCGGAGACCCCTTAGATCGA<br>R: TAGCCTGTAAAAGATTTCTGCAAACC	56
CPT1	F: GGTTGCTGATGACGGCTATGGTGT<br>R: GCGGTGAGGCCAAACAAGGTGATA	60
ACLY	F: GCCAGCGGGAGCACATC<br>R: CTTTGCAGGTGCCACTTCATC	62
MCAD	F: AACACTTACTATGCCTCGATTGCA<br>R: CCATAGCCTCCGAAAATCTGAA	58
Ywhaz	F: GAAAAGTTCTTGATCCCCAATGC<br>R: TGTGACTGGTCCACAATTCCTT	60

2. Supplementary Figures

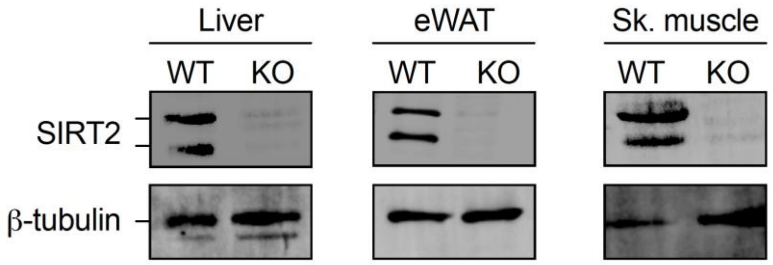

Figure S1: Absence of SIRT2 expression in multiple tissues from SIRT2-KO mice**.** Representative Western blots showing SIRT2 expression in liver, epididymal adipose tissue (eWAT), and skeletal muscle (Sk. muscle) from WT and SIRT2-KO mice fed a CD. Tubulin was used as a loading control; protein levels were normalized to the tubulin levels in the same lane.

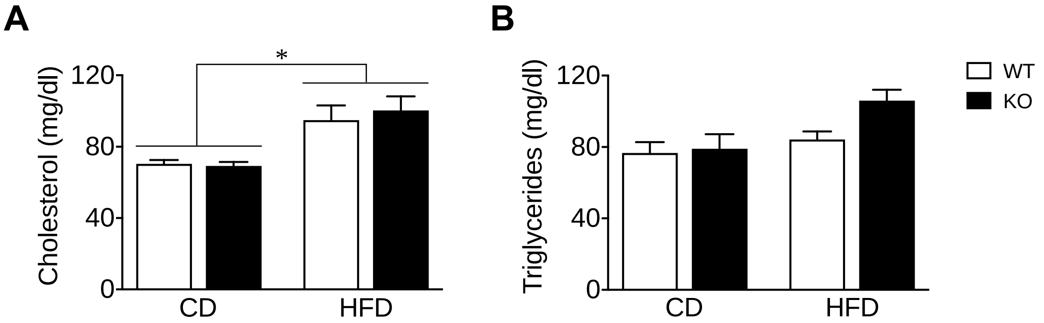

Figure S2: Serum levels of cholesterol and triglycerides. Serum concentration of (**A**) total cholesterol and (**B**) triglyceride levels of WT and SIRT2-KO mice fed a CD or a HFD at the end of the dietary intervention measured after 15 hours of fasting. Data are presented as mean ± SEM; n= 4-8 per group; *P < 0.05.

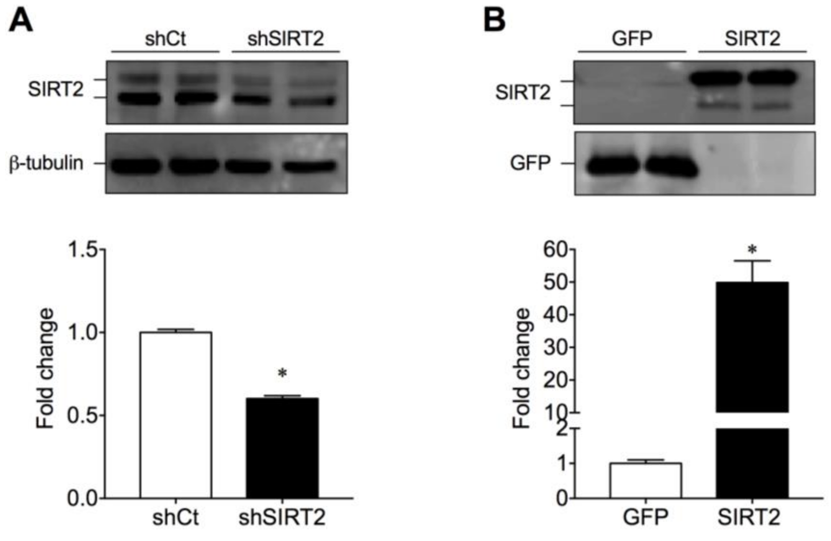

Figure S3: Efficiency of SIRT2 silencing and overexpression in HepG2 cells**.** (**A**) Representative Western blots and quantification showing SIRT2 expression in HepG2 cells transduced with lentivirus to silence SIRT2 (shSIRT2) and the control (shCT). Tubulin was used as a loading control; protein levels were normalized to the tubulin levels in the same lane. (**B**) Representative Western blots and quantification showing SIRT2 expression in HepG2 cells transduced with lentivirus to overexpress SIRT2 (SIRT2) and the control (GFP). Data are presented as mean ± SEM; n= 2 per condition; *P < 0.05.
