# Supplementary figures and images for: SIRT2 Deficiency Exacerbates Hepatic Steatosis via a Putative Role of the ER Stress Pathway
Source: Int J Mol Sci. 2022 Jun 17;23(12):6790. doi: 10.3390/ijms23126790 (PMC9223775; doi:10.3390/ijms23126790)

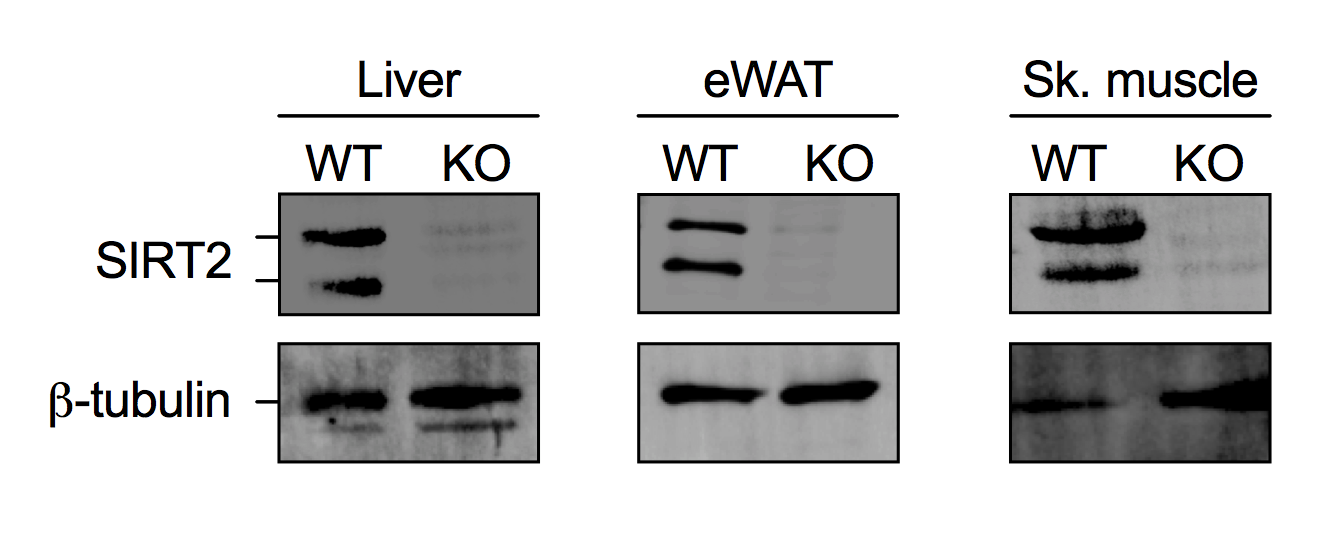

Supplement: Supplementary file 1 [file ijms-23-06790-s001.zip › ijms-1707054-supplementary/ijms-1707054-supplementary/ijms-1707054-Supplementary Figure S1.tiff]

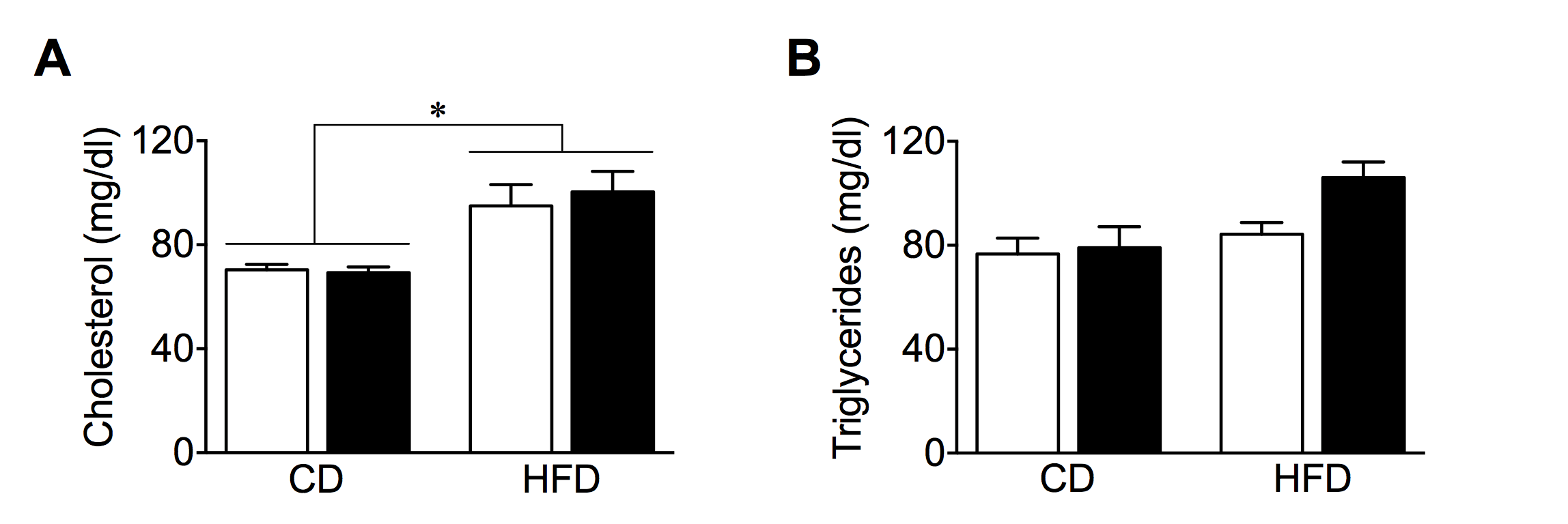

Supplement: Supplementary file 1 [file ijms-23-06790-s001.zip › ijms-1707054-supplementary/ijms-1707054-supplementary/ijms-1707054-Supplementary Figure S2.tiff]

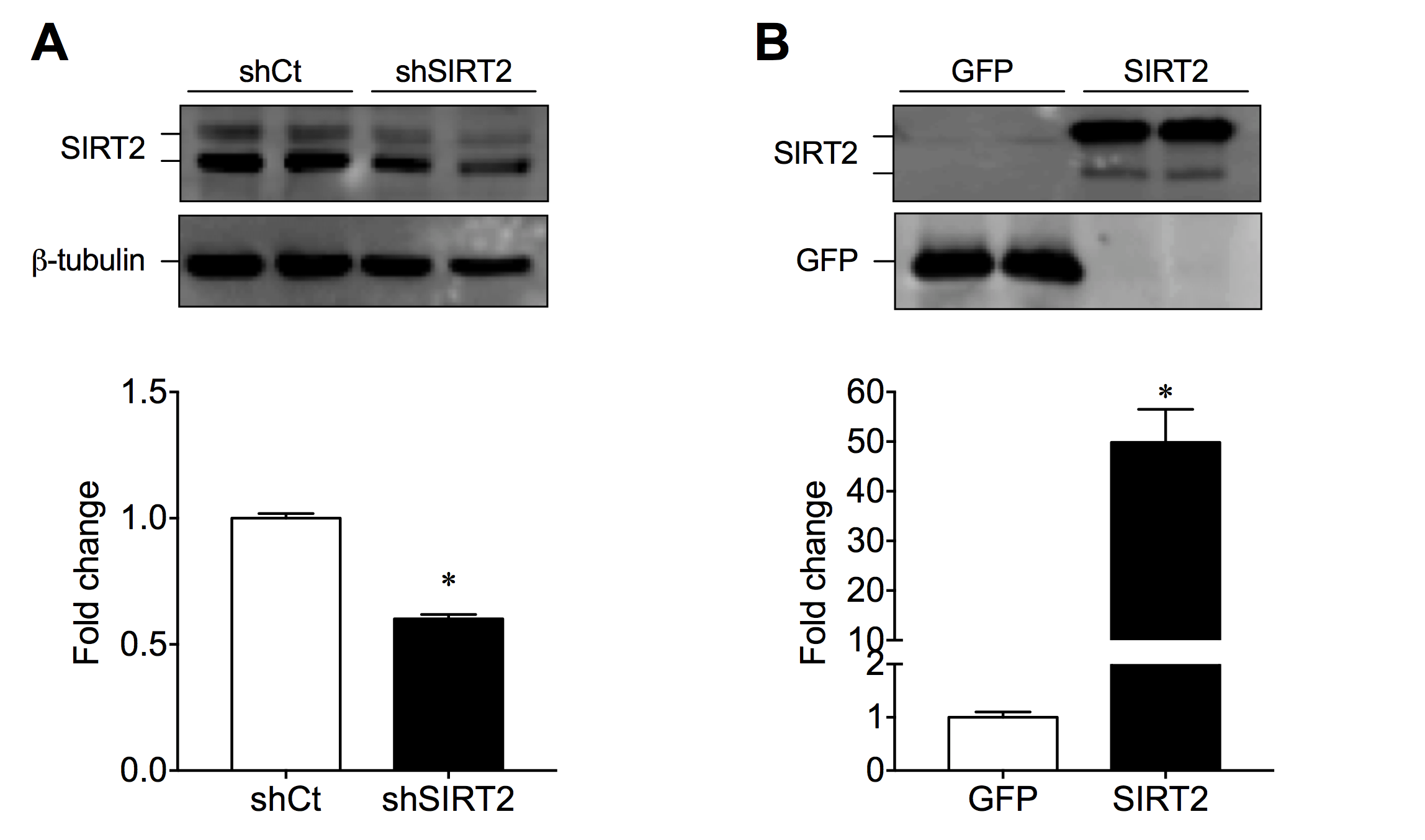

Supplement: Supplementary file 1 [file ijms-23-06790-s001.zip › ijms-1707054-supplementary/ijms-1707054-supplementary/ijms-1707054-Supplementary Figure S3.tiff]
